# How Is One's Own and (Ex‐)Partner's Professional Prestige Associated With Loneliness and Isolation? Findings Based on Cross‐Sectional Data From Middle‐Aged and Older Adults Living in Germany in 2022/2023

**DOI:** 10.1002/hsr2.71371

**Published:** 2025-10-13

**Authors:** André Hajek, Larissa Zwar, Razak M. Gyasi, Dong Keon Yon, Hans Oh, Pinar Soysal, Supa Pengpid, Karl Peltzer, Hans‐Helmut König

**Affiliations:** ^1^ Department of Health Economics and Health Services Research University Medical Center Hamburg‐Eppendorf, Hamburg Center for Health Economics Hamburg Germany; ^2^ Department of Psychology Brock University St. Catharines Ontario Canada; ^3^ African Population and Health Research Center Nairobi Kenya; ^4^ National Centre for Naturopathic Medicine, Faculty of Health Southern Cross University Lismore New South Wales Australia; ^5^ Center for Digital Health, Medical Science Research Institute Kyung Hee University College of Medicine Seoul South Korea; ^6^ Suzanne Dworak Peck School of Social Work University of Southern California Los Angeles California USA; ^7^ Department of Geriatric Medicine, Faculty of Medicine Bezmialem Vakif University Istanbul Turkiye; ^8^ Department of Health Education and Behavioral Sciences, Faculty of Public Health Mahidol University Bangkok Thailand; ^9^ Department of Public Health Sefako Makgatho Health Sciences University Pretoria South Africa; ^10^ Department of Healthcare Administration, College of Medical and Health Science Asia University Taichung Taiwan; ^11^ Department of Psychology University of the Free State Bloemfontein South Africa; ^12^ Department of Medical Research China Medical University Hospital, China Medical University Taichung Taiwan

**Keywords:** loneliness, occupational prestige, occupational reputation, professional esteem, professional prestige, professional reputation, social isolation

## Abstract

**Background and Aims:**

Thus far, very little is known about the association between occupational prestige and loneliness. Thus, our aim was to investigate how (1) one's own professional prestige, (2) the professional prestige of one's partner and (3) the professional standing of one's last spouse (for those without a spouse) are associated with loneliness and isolation.

**Methods:**

Data were used from the most recent wave of the nationally representative German Ageing Survey including individuals aged 43 years and above residing in private settings (wave 8, *n* = 3059 individuals; average age was 67.0 years, 52.2% female). Psychometrically sound instruments were used to measure the key variables. Sociodemographic, lifestyle‐ and health‐related covariates were added to the linear regression model.

**Results:**

Higher own professional prestige was associated with lower loneliness and social isolation scores. In contrast, the professional prestige of one's partner and the professional standing of the last spouse (for those without a spouse) were both not significantly associated with loneliness and social isolation.

**Conclusion:**

Data suggest that one's own favorable professional prestige was associated with lower loneliness and social isolation levels, whereas the professional prestige of the (ex‐)partner was unrelated to such social factors. Future longitudinal studies should analyze whether changing to a job with more professional prestige might be associated with changes in loneliness and social isolation.

## Summary

- Recent data were taken from the German Ageing Survey
- How is one's own and (ex‐)partner's professional prestige associated with loneliness and isolation?
- Higher own professional prestige was associated with lower loneliness and social isolation scores
- In contrast, the professional prestige of one's partner and the professional standing of the last spouse (for those without a spouse) were both not significantly associated with loneliness and social isolation
- This knowledge helps to identify individuals at risk of loneliness and isolation

## 1 Introduction

Ongoing demographic change is taking place in many countries worldwide. It is projected that the number of older individuals will markedly increase in the next decades in numerous countries. In old age, several critical life events can take place such as falls, losing relatives and friends, and severe health deteriorations. These and other factors may contribute to feelings of loneliness (difference between actual and desired social relationships [1]) and perceived social isolation (feeling of not belonging to the society [2]) in later life [3, 4, 5]. Investigating the factors linked to loneliness and social isolation is important because such unmet social needs can lead to chronic illnesses and premature death [6, 7, 8, 9].

Thus far, several factors associated with loneliness and social isolation among middle‐aged and older adults have been identified. For example, support services such as caring for grandchildren, volunteering, or dog ownership, may help to reduce loneliness and social isolation [10, 11, 12, 13]. Avoiding poverty or maintaining health can be important for reducing loneliness and isolation [14, 15]. Other studies showed that education was mostly unrelated to loneliness [16]. Some studies have also examined the relationship of work‐related factors with loneliness and isolation (as an overview [16]). However, they mostly focused on general work‐related factors such as unemployment, showing an association with higher loneliness/isolation [16]. Thus far, very little is known about the association between occupational prestige and loneliness. In fact, one single study [17] showed that individuals with medium, low, and very low occupational prestige had higher loneliness scores compared to individuals with very high occupational prestige based on German data from 2014. This former study attributed this association to work‐related social networks and social status [17]. The previous study, however, focused solely on participants' own professional prestige and loneliness. There is therefore a complete lack of studies that also look at the professional prestige of the partner or former partner in association with loneliness (and also in association with social isolation). This is a notable omission given that partnered individuals tend to share social networks to a large degree. In light of this gap, the aim of this study was to investigate how (1) one's own professional prestige, (2) the professional prestige of one's partner and (3) the professional standing of one's last spouse (for those without a spouse) is associated with loneliness and isolation. This present study can significantly advance our understanding of work‐related factors—such as professional prestige—in association with loneliness and isolation, which are linked to adverse health consequences and successful aging.

Following the previous study [17], we hypothesize that higher professional prestige is significantly associated with lower loneliness and isolation. We expect that one's own prestige in particular is significantly associated with loneliness and isolation (due to the link between professional prestige and self‐worth [18]), but we also predict that partner's prestige could be relevant for loneliness and isolation due to spillover effects. We cautiously assume that the prestige of the last spouse may no longer be relevant for loneliness and social isolation.

## 2 Methods

### 2.1 Sample

Data were drawn from the most recent wave 8 of the German Ageing Survey (“Deutscher Alterssurvey”, DEAS), with data collection taking place between December 2022 and June 2023. We focused on data from wave 8 because we were interested in investigating the associations of interest with the latest data. Interviews were conducted via telephone (CATI) and in person (CAPI). The response rate was about 62%. Only a slight selection bias was identified. For example, participation rates were higher among younger individuals, those with higher education, women, and individuals in better health, whereas factors such as family situation, size of the region or income were mainly not associated with such rates. The interviews lasted around 81 min.

After completing the interview, participants could fill out an additional questionnaire (by post or online) addressing more sensitive issues such as loneliness and social isolation.

As a token of appreciation, all participants received a letter of thanks and a small gift of €15. All individuals participating in the DEAS study provided informed consent. The study adhered to the Declaration of Helsinki and its subsequent amendments. An ethical review was not required as the study did not meet the criteria for such a review (e.g., it did not involve examination of patients or pose risks to participants).

### 2.2 Outcomes: Loneliness and Social Isolation

The well‐established De Jong Gierveld tool [19] was employed to assess overall feelings of loneliness. The 6‐item version was used. Thereof, three items focus on emotional loneliness and three items on social loneliness. Before averaging the six items to obtain a final score, three of these items were recoded. The resulting score ranges from 1 to 4, whereby higher values indicate higher levels of overall loneliness. In this study, the reliability of the scale was confirmed (Cronbach's alpha and McDonald's omega were both 0.84).

The Bude and Lantermann instrument [2], which comprises four items, was used to evaluate social isolation. A final score was determined by averaging the responses across all four items. This final score ranges from 1 to 4, with higher values indicating higher levels of social isolation. In this study, the reliability of the measure was demonstrated (Cronbach's alpha and McDonald's omega were both 0.87).

### 2.3 Independent Variables of Interest

A recently developed tool was used to measure professional prestige (German language: “Berufliche Ansehensskala”, in short: BAS). Since the provision of data from wave 8 of the DEAS study, this scale has been available in this study. This tool is available for the German Classification of Occupations 2010 (German language, “Klassifikation der Berufe 2010”, in short: KldB 2010 [20]), developed by the Federal Employment Agency and the Institute for Employment Research (among other things, with help of the Federal Statistical Office). It is highly compatible with international classifications such as the International Standard Classification of Occupations 2008 (ISCO‐08) [21].

Similar to US scales [22], reputation was measured using a response format with 11 categories ranging from 0 (lowest professional prestige) to 10 (highest professional prestige). Thus, society assigns values to jobs. Examples for prestigious jobs in Germany are chief physicians, professors, or engineers in research and development. Examples for non‐prestigious jobs are: taxi drivers, clothing salesmen or building cleaners.

Reputation values were collected for more than 400 occupations. To this end, 9011 individuals were interviewed by computer‐assisted telephone (representative sample). Data collection took place from October 2017 to May 2018. The former study also demonstrated high reliability and the validity of the BAS [23]. The development and more details about the psychometric properties of the BAS is described in further detail in Ebner and Rohrbach‐Schmidt [24].

The professional prestige was available for (1) respondents, (2) their partners (married and unmarried), and (3) the last spouses (i.e., only married) of respondents currently without a partner. It is of note that, for example, when individuals retired, their professional prestige was based on their last job (the same applies for their partners).

### 2.4 Covariates

In line with previous research [4, 25, 26, 27], a broad set of covariates was included into regression analysis. The sociodemographic factors considered included sex (male, female), marital status (married and living with spouse, married but separated, single, widowed, divorced), employment status (employed, retired, and other), as well as education level (classified according to the ISCED framework [28], which encompasses three categories: low, medium, and high education).

Concerning lifestyle‐related factors, adjustments were made for the frequency of physical activity, categorized as follows: daily, several times a week, once a week, 1–3 times a month, less often, and never. Alcohol consumption was examined with the same response categories (i.e., from daily to never).

In terms of health‐related factors, adjustments were made for probable depression, physical functioning, and the presence of chronic conditions. Probable depression was assessed using the 15‐item version of the Center for Epidemiologic Studies Depression Scale [29], yielding a sum score from 0 to 45 (where higher scores indicate more depressive symptoms). Individuals were classified as having probable depression if their score was at least 18. Physical functioning was measured using the physical functioning subscale of the SF‐36 [30]. The resulting total score ranges from 0 to 100, with higher scores indicating more favorable physical functioning. Additionally, a count score of chronic conditions was calculated based on the presence or absence of 11 chronic conditions (cardiac and circulatory disorders; bad circulation; joint, bone, spinal or back problems; respiratory problems, asthma, shortness of breath; stomach and intestinal problems; cancer; diabetes; gall bladder, liver or kidney problems; bladder problems; eye problems, vision impairment; ear problems, hearing problems).

### 2.5 Statistical Analysis

Sample characteristics are first shown. Thereafter, unadjusted and adjusted linear regressions are computed. Loneliness and social isolation were used as outcome measures. In a robustness check, ordered probit regressions (rather than linear regressions) were used. Professional prestige for respondents, for their partners and for the last spouses (for individuals currently without a partner) served as key independent variables. It is worth repeating that it was adjusted for age, sex, marital status, employment status, education, frequency of sports activity, alcohol intake, number of chronic conditions, physical functioning, and probable depression. Robust standard errors were computed. Exploratory moderation analyses were conducted examining the interaction of key sociodemographic factors (i.e., age, sex and education) and coping mechanisms (general self‐efficacy, based on the 5‐item general self‐efficacy scale [31, 32] and general self‐esteem, based on the 10‐item Rosenberg scale [33], see also [34, 35]) with professional prestige for respondents. Since the proportion of missings was low, listwise deletion was used in regression analysis to deal with missing data. Sampling weights were not applied.

McDonald's omega was calculated based on a tool developed by Shaw [36]. Statistical significance was determined with a *p* value under 0.05 (tests were two‐sided), and analyses were performed using StataNow MP 18.5 software (Stata Corp., College Station, Texas).

## 3 Results

### 3.1 Sample Characteristics

The characteristics of the analytic sample (*n *= 3059 individuals) are described in Table 1. The mean age was 67.0 years (standard deviation, SD: 10.7 years; 43–95 years), with 52.2% of the respondents being female. Most of the individuals (68.6%) were married, living together with spouse. Mean loneliness was 1.7 (SD: 0.5) and mean social isolation was 1.6 (SD: 0.6). The mean professional prestige for respondents was 5.8 (SD: 0.8; from 4.2 to 8.1). Similarly, the mean professional prestige for partners was 5.7 (SD: 0.8, also from 4.2 to 8.1) and mean professional prestige for last spouses was 5.7 (SD: 0.8, from 4.2 to 7.8). More details are shown in Table 1.

**Table 1:** Characteristics of the sample, with *n* = 3059 individuals.

Variables	Mean (SD)/*n* (%)
Age	67.0 (10.7)
Sex	
Men	1463 (47.8%)
Women	1596 (52.2%)
Marital status	
Married, living together with spouse	2098 (68.6%)
Married, living separated from spouse	34 (1.1%)
Divorced	313 (10.2%)
Widowed	368 (12.0%)
Single (never married)	246 (8.0%)
Education	
Low	86 (2.8%)
Medium	1400 (45.8%)
High	1573 (51.4%)
Employment status	
Employed	932 (30.5%)
Retired	1976 (64.6%)
Other: not employed	151 (4.9%)
Sports activity (frequency)	
Daily	301 (9.8%)
Several times a week	1014 (33.1%)
Once a week	531 (17.4%)
1–3 times a month	186 (6.1%)
Less often	312 (10.2%)
Never	715 (23.4%)
Alcohol intake (frequency)	
Daily	315 (10.3%)
Several times a week	808 (26.4%)
Once a week	513 (16.8%)
1–3 times a month	418 (13.7%)
Less often	693 (22.7%)
Never	312 (10.2%)
Probable depression	
Absence	2872 (93.9%)
Presence	187 (6.1%)
Count of chronic conditions (from 0 to 11)	2.6 (2.0)
Physical functioning (from 0 to 100; with higher scores corresponding to better physical functioning)	83.0 (21.5)
Loneliness (1–4, with higher values corresponding to higher loneliness levels)	1.7 (0.5)
Social isolation (1–4, with higher values corresponding to higher social isolation levels)	1.6 (0.6)
Professional prestige for respondents (from 0 to 10, with higher values corresponding to higher prestige)	5.8 (0.8)
Professional prestige for partners (from 0 to 10, with higher values corresponding to higher prestige)	5.7 (0.8)
Professional prestige for last spouses (when respondents are currently without a partner) (from 0 to 10, with higher values corresponding to higher prestige)	5.7 (0.8)

### 3.2 Regression Analysis

Results of linear regressions for the associations of professional prestige with loneliness and social isolation (both unadjusted and adjusted) are presented in Tables 2 and 3, respectively.

**Table 2:** Professional prestige and loneliness. Results of linear regressions (wave 8).

	Loneliness (unadjusted)	Loneliness (unadjusted)	Loneliness (unadjusted)	Loneliness (adjusted)	Loneliness (adjusted)	Loneliness (adjusted)
Professional prestige for respondents	−0.05***			−0.02*		
	(−0.07 to −0.02)			(−0.05 to −0.00)		
Professional prestige for partners		−0.04***			−0.01	
		(−0.07 to −0.02)			(−0.04 to 0.01)	
Professional prestige for last spouses (when respondents are currently without a partner)			−0.01			0.01
			(−0.08 to 0.06)			(−0.07 to 0.08)
Covariates				✓	✓	✓
Individuals	3141	2611	420	3059	2544	406
*R*²	0.005	0.004	0.0001	0.11	0.10	0.13

*Note:* Unstandardized beta coefficients are presented. 95% CI in parentheses; +*p* < 0.10. Covariates include: age, sex, marital status, employment status, education, frequency of sports activity, alcohol intake, number of chronic conditions, physical functioning, and probable depression. The sample sizes varied for two key reasons (1): the number of missings in the outcomes and (2) the fact that some respondents did not have a partner or ex‐spouse.

* *p* < 0.05

** *p* < 0.01

*** *p* < 0.001.

**Table 3:** Professional prestige and social isolation. Results of linear regressions (wave 8).

	Social isolation (unadjusted)	Social isolation (unadjusted)	Social isolation (unadjusted)	Social isolation (adjusted)	Social isolation (adjusted)	Social isolation (adjusted)
Professional prestige for respondents	−0.07***			−0.03**		
	(−0.09 to −0.04)			(−0.06 to −0.01)		
Professional prestige for partners		−0.05***			−0.02	
		(−0.08 to −0.03)			(−0.04 to 0.01)	
Professional prestige for last spouses (when respondents are currently without a partner)			−0.06+			−0.03
			(−0.13 to 0.01)			(−0.09 to 0.04)
Covariates				✓	✓	✓
Individuals	3146	2604	424	3060	2536	409
*R*²	0.01	0.01	0.01	0.14	0.14	0.13

*Note:* Unstandardized beta coefficients are presented. 95% CI in parentheses; Covariates include: age, sex, marital status, employment status, education, frequency of sports activity, alcohol intake, number of chronic conditions, physical functioning, and probable depression. The sample sizes varied for two key reasons (1): the number of missings in the outcomes and (2) the fact that some respondents did not have a partner or ex‐spouse.

+ *p* < 0.10;

* *p* < 0.05

** *p* < 0.01

*** *p* < 0.001.

In adjusted regressions, higher professional prestige for respondents was significantly associated with lower loneliness levels (*β* = −0.02, *p* < 0.05). In contrast, professional prestige for partners and professional prestige for last spouses were not significantly associated with loneliness in adjusted regressions.

Higher professional prestige for respondents was also significantly associated with lower social isolation levels (*β* = −0.03, *p* < 0.01), whereas professional prestige for partners and professional prestige for last spouses were not significantly associated with social isolation in adjusted regressions.

It is worth noting that the associations of professional prestige for respondents with both outcomes were not significantly moderated by age, sex, education, self‐efficacy or self‐esteem (all ns; further details (including covariates) are shown in Supporting Information S1). For example, the conditional main effect

Of note that higher own professional prestige was also associated with lower loneliness and social isolation scores when it was additionally adjusted for household net income. When ordered probit regressions rather than linear regressions were used, the results remained virtually the same in terms of significance.

## 4 Discussion

Using data from a large, nationally representative sample, our aim was to examine how one's own professional prestige, the professional prestige of one's partner, and the professional prestige of the last spouse (for those without a spouse) is associated with loneliness and isolation. Our key findings were as follows: Higher own professional prestige was associated with lower loneliness and social isolation scores (such associations were not moderated by age, sex and education), even after adjusting for various covariates (such as socioeconomic or health‐related factors). In contrast, the professional prestige of one's spouse and the professional standing of the last spouse (for those without a spouse) were both not significantly associated with loneliness and social isolation. However, it is worth noting that the effect sizes (in terms of partial eta²) for such associations were very small. Our current study markedly adds to our present knowledge by (1) examining the professional prestige comprehensively (i.e., one's own, one's partner's and one's former spouse's), (2) by investigating both loneliness and social isolation, and (3) by using very recent data from a large nationally representative sample.

One comparable previous German study [17] also showed an association between one's own lower professional prestige and higher loneliness scores, supporting our present findings. Other comparable studies are currently not available. With regard to potential explanations, most apparently, one's own professional roles or prestige may have a direct effect on general self‐esteem [18], which in turn impacts social interactions and the formation of social relationships. In other words: Low self‐esteem related to one's professional lack of prestige may contribute to higher loneliness and isolation levels [37]. Moreover, classism may also contribute to lower self‐worth [38]. For instance, when other individuals disparage somebody with low professional prestige, such individuals may develop a negative self‐image [39]. This can lead to social withdrawal ultimately contributing to loneliness and isolation [40]. Furthermore, when individuals with low professional prestige compare themselves with other individuals with higher professional prestige, they may develop a deleterious self‐image [41]. Moreover, individuals with high professional prestige may have a higher meaning in life which could contribute to lower loneliness and isolation scores.

Interestingly, the association of one's own professional prestige with loneliness and social isolation was not significantly moderated by sex, age or education ‐ suggesting that the associations of interest were similar across sociodemographic groups in this sample. Given the fact that previous research often stressed the importance of work for psychosocial outcomes among men [42], such findings (especially regarding sex) are a bit surprising. Perhaps these results primarily show that the professional prestige of one's own job is not only important for men, but also for women (see also [43]. However, future research from other countries is needed to compare our current results with. Moreover, the lack of moderation with age can also show that professional prestige (of the current and previous job—e.g., if retired) can still be relevant for loneliness and isolation even when individuals are no longer working. This stresses the role of the profession and the associated prestige for social unmet needs. In future work, a comparison with other countries where a large proportion of individuals continue to work well into old age (e.g., South Korea or Japan) would be of interest. It is worth noting that the “prestige” of a given occupation is socially assigned and designated within a social hierarchy.

The professional prestige of one's partner and the professional prestige of the last spouse were not significantly associated with loneliness and social isolation in our study. Potential explanations may refer to the fact that the professional prestige of such partners are presumably not relevant for one's own self‐worth. Apparently, for many individuals, this prestige is not a reason to withdraw socially or to avoid contact with friends, relatives or job colleagues, suggesting no spillover effects. We assume that the quality of the relationships (e.g., of the partner with other friends) is probably much more important for one's own loneliness and isolation.

Some strengths and limitations of this study are worth noting. First, data were taken from a large, nationally representative sample of community‐dwelling individuals aged 43 years and over. Sound tools were used to quantify both outcomes and the key independent variables. Several covariates were added to the regression model. A limitation of the data is its cross‐sectional design. Thus, we cannot dismiss the possibility that increases in loneliness lead to changes in one's own professional prestige. However, we think that the direction from professional prestige to loneliness and isolation is much more reasonable. Moreover, it should be noted that the associations identified (of professional prestige with loneliness and social isolation) are small.

In summary, data suggest that one's own favorable professional prestige was associated with lower loneliness and social isolation levels, whereas the professional prestige of the (ex‐)partner is unrelated to such social factors. Globally speaking, very little is known about work‐related factors (professional prestige in particular) and loneliness/isolation in old age [16]. Therefore, we would like to encourage further research: Future longitudinal studies should analyze whether changing to a job with more professional prestige might be beneficial for these social factors. Future research in other areas of the world would also be desirable. In other countries where people define themselves particularly strongly through their work (such as South Korea, Japan, or the United States), this association of interest may be more pronounced. Findings from caste‐dominated India would also be of interest. Moreover, future work can distinguish between self‐employed and employed individuals – in their relationship with loneliness and isolation.

## Author Contributions

**André Hajek:** conceptualization, data curation, formal analysis, methodology, project administration, visualization, writing – original draft. **Larissa Zwar:** conceptualization, visualization, writing – review and editing. **Razak M Gyasi:** conceptualization, visualization, writing – review and editing. **Dong Keon Yon:** conceptualization, visualization, writing – review and editing. **Hans Oh:** conceptualization, visualization, writing – review and editing. **Pinar Soysal:** conceptualization, visualization, writing – review and editing. **Supa Pengpid:** conceptualization, visualization, writing – review and editing. **Karl Peltzer:** conceptualization, visualization, writing – review and editing. **Hans‐Helmut König:** conceptualization, resources, supervision, visualization, writing – review and editing.

## Ethics Statement

All individuals participating in the DEAS study provided informed consent. The study adhered to the Declaration of Helsinki and its subsequent amendments. An ethical review was not required as the study did not meet the criteria for such a review (e.g., it did not involve examination of patients or pose risks to participants).

## Conflicts of Interest

The authors declare no conflicts of interest.

## Transparency Statement

The lead author, André Hajek, affirms that this manuscript is an honest, accurate, and transparent account of the study being reported; that no important aspects of the study have been omitted; and that any discrepancies from the study as planned (and, if relevant, registered) have been explained.

## Supporting information

Supporting File 1.

## Data Availability

The data used in this study are third‐party data. The anonymized data sets of the DEAS (1996, 2002, 2008, 2011, 2014, 2017, 2020, 2020/2021, 2023) are available for secondary analysis. The data has been made available to scientists at universities and research institutes exclusively for scientific purposes. The use of data is subject to written data protection agreements. Microdata of the German Ageing Survey (DEAS) are available free of charge to scientific researchers for non‐profitable purposes. The FDZ‐DZA provides access and support to scholars interested in using DEAS for their research. However, for reasons of data protection, signing a data distribution contract is required before data can be obtained. For further information on the data distribution contract, please see https://www.dza.de/en/research/fdz/access-to-data/application (accessed on April 8, 2025).
