# Supplementary material for: How Is One's Own and (Ex‐)Partner's Professional Prestige Associated With Loneliness and Isolation? Findings Based on Cross‐Sectional Data From Middle‐Aged and Older Adults Living in Germany in 2022/2023
Source: Health Sci Rep. 2025 Oct 13;8(10):e71371. doi: 10.1002/hsr2.71371 (PMC12518505; doi:10.1002/hsr2.71371)
Supplement: Supplementary file 1 — Supporting File 1. [file HSR2-8-e71371-s001.docx]

Supplementary Table 1. Professional prestige and loneliness. Results of linear regressions (wave 8) (all covariates are displayed; interaction terms are also shown)

|  |  |  |  |  |  |  |  |  |
| --- | --- | --- | --- | --- | --- | --- | --- | --- |
|  | Loneliness | Loneliness | Loneliness | Loneliness | Loneliness | Loneliness | Loneliness | Loneliness |
|  |  |  |  |  |  |  |  |  |
|  |  |  |  |  |  |  |  |  |
| Professional prestige for respondents | -0.02* |  |  | -0.02 | -0.14+ | -0.10 | 0.02 | 0.05 |
|  | (-0.05 - -0.00) |  |  | (-0.05 - 0.01) | (-0.29 - 0.01) | (-0.25 - 0.05) | (-0.16 - 0.20) | (-0.10 - 0.21) |
|  |  |  |  |  |  |  |  |  |
| Professional prestige for partners |  | -0.01 |  |  |  |  |  |  |
|  |  | (-0.04 - 0.01) |  |  |  |  |  |  |
| Professional prestige for last spouses (when respondents are currently without a partner) |  |  | 0.01 |  |  |  |  |  |
|  |  |  | (-0.07 - 0.08) |  |  |  |  |  |
|  |  |  |  |  |  |  |  |  |
| Interaction terms: |  |  |  |  |  |  |  |  |
|  |  |  |  |  |  |  |  |  |
| Women x Professional prestige for respondents |  |  |  | -0.01 |  |  |  |  |
|  |  |  |  | (-0.06 - 0.04) |  |  |  |  |
| Age x Professional prestige for respondents |  |  |  |  | 0.00 |  |  |  |
|  |  |  |  |  | (-0.00 - 0.00) |  |  |  |
| Medium education x Professional prestige for respondents |  |  |  |  |  | 0.07 |  |  |
|  |  |  |  |  |  | (-0.08 - 0.23) |  |  |
| High education x Professional prestige for respondents |  |  |  |  |  | 0.08 |  |  |
|  |  |  |  |  |  | (-0.07 - 0.23) |  |  |
| Self-esteem x Professional prestige for respondents |  |  |  |  |  |  | -0.01 |  |
|  |  |  |  |  |  |  | (-0.06 - 0.04) |  |
| Self-efficacy x Professional prestige for respondents |  |  |  |  |  |  |  | -0.02 |
|  |  |  |  |  |  |  |  | (-0.07 - 0.03) |
| Covariates |  |  |  |  |  |  |  |  |
|  |  |  |  |  |  |  |  |  |
| Age | -0.00** | -0.00* | -0.01* | -0.00** | -0.01* | -0.00** | -0.00*** | -0.01*** |
|  | (-0.01 - -0.00) | (-0.01 - -0.00) | (-0.02 - -0.00) | (-0.01 - -0.00) | (-0.03 - -0.00) | (-0.01 - -0.00) | (-0.01 - -0.00) | (-0.01 - -0.00) |
| Sex: Women (Reference category: Men) | -0.13*** | -0.11*** | -0.18** | -0.07 | -0.13*** | -0.13*** | -0.08*** | -0.13*** |
|  | (-0.17 - -0.09) | (-0.15 - -0.06) | (-0.30 - -0.05) | (-0.34 - 0.20) | (-0.17 - -0.09) | (-0.17 - -0.09) | (-0.12 - -0.05) | (-0.17 - -0.10) |
| Marital status: - Married, living separated from spouse (Reference category: Married, living together with spouse) | 0.21* | 0.13 |  | 0.21* | 0.21* | 0.21* | 0.09 | 0.17* |
|  | (0.04 - 0.38) | (-0.12 - 0.38) |  | (0.04 - 0.38) | (0.04 - 0.38) | (0.04 - 0.38) | (-0.07 - 0.24) | (0.01 - 0.33) |
| - Divorced | 0.10** | 0.01 | 0.01 | 0.10** | 0.10** | 0.10** | 0.10*** | 0.11*** |
|  | (0.03 - 0.17) | (-0.08 - 0.11) | (-0.24 - 0.25) | (0.03 - 0.17) | (0.03 - 0.17) | (0.03 - 0.17) | (0.05 - 0.16) | (0.05 - 0.17) |
| - Widowed | 0.04 | -0.10+ | -0.04 | 0.04 | 0.04 | 0.03 | 0.04 | 0.06+ |
|  | (-0.03 - 0.10) | (-0.20 - 0.00) | (-0.28 - 0.19) | (-0.03 - 0.10) | (-0.02 - 0.10) | (-0.03 - 0.10) | (-0.02 - 0.09) | (-0.00 - 0.11) |
| - Single | 0.20*** | 0.10+ | -0.00 | 0.20*** | 0.20*** | 0.20*** | 0.12** | 0.15*** |
|  | (0.12 - 0.28) | (-0.01 - 0.21) | (-0.36 - 0.36) | (0.12 - 0.28) | (0.12 - 0.28) | (0.12 - 0.28) | (0.05 - 0.19) | (0.08 - 0.23) |
| Employment status: - Retired (Reference category: Employed) | 0.01 | -0.00 | 0.07 | 0.01 | 0.01 | 0.01 | 0.00 | 0.01 |
|  | (-0.05 - 0.07) | (-0.07 - 0.06) | (-0.14 - 0.27) | (-0.05 - 0.07) | (-0.05 - 0.07) | (-0.05 - 0.07) | (-0.05 - 0.06) | (-0.05 - 0.07) |
| - Other: not employed | 0.04 | 0.02 | 0.01 | 0.04 | 0.04 | 0.04 | 0.01 | 0.05 |
|  | (-0.06 - 0.14) | (-0.09 - 0.13) | (-0.27 - 0.28) | (-0.06 - 0.14) | (-0.06 - 0.14) | (-0.06 - 0.14) | (-0.07 - 0.10) | (-0.04 - 0.14) |
| Education: - Medium education (Reference category: Low education) | -0.05 | -0.12 | 0.03 | -0.05 | -0.05 | -0.43 | 0.01 | -0.03 |
|  | (-0.16 - 0.07) | (-0.26 - 0.02) | (-0.20 - 0.26) | (-0.16 - 0.07) | (-0.16 - 0.06) | (-1.26 - 0.40) | (-0.10 - 0.11) | (-0.14 - 0.08) |
| - High education | -0.07 | -0.15* | -0.02 | -0.06 | -0.07 | -0.48 | 0.02 | -0.04 |
|  | (-0.18 - 0.05) | (-0.29 - -0.01) | (-0.27 - 0.23) | (-0.18 - 0.05) | (-0.18 - 0.05) | (-1.32 - 0.35) | (-0.08 - 0.13) | (-0.15 - 0.07) |
| Frequency of sports activities: - Several times per week (Reference category: Daily) | 0.02 | 0.04 | 0.04 | 0.02 | 0.02 | 0.02 | 0.02 | 0.01 |
|  | (-0.04 - 0.09) | (-0.03 - 0.11) | (-0.13 - 0.21) | (-0.04 - 0.09) | (-0.04 - 0.09) | (-0.04 - 0.09) | (-0.04 - 0.07) | (-0.05 - 0.07) |
| - Once a week | 0.03 | 0.01 | -0.02 | 0.03 | 0.03 | 0.03 | -0.01 | 0.01 |
|  | (-0.04 - 0.10) | (-0.07 - 0.09) | (-0.20 - 0.16) | (-0.04 - 0.10) | (-0.04 - 0.10) | (-0.04 - 0.10) | (-0.07 - 0.06) | (-0.06 - 0.07) |
| - 1-3 times a month | -0.01 | -0.02 | 0.04 | -0.01 | -0.01 | -0.01 | -0.03 | -0.02 |
|  | (-0.11 - 0.08) | (-0.12 - 0.08) | (-0.29 - 0.36) | (-0.11 - 0.08) | (-0.11 - 0.08) | (-0.11 - 0.08) | (-0.11 - 0.05) | (-0.11 - 0.07) |
| - Less often | 0.05 | 0.06 | 0.14 | 0.05 | 0.05 | 0.05 | 0.03 | 0.05 |
|  | (-0.03 - 0.13) | (-0.03 - 0.14) | (-0.12 - 0.40) | (-0.03 - 0.13) | (-0.03 - 0.13) | (-0.03 - 0.13) | (-0.04 - 0.10) | (-0.03 - 0.12) |
| - Never | 0.07* | 0.08+ | 0.02 | 0.08* | 0.07* | 0.07* | 0.03 | 0.05 |
|  | (0.00 - 0.15) | (-0.00 - 0.16) | (-0.14 - 0.18) | (0.00 - 0.15) | (0.00 - 0.14) | (0.00 - 0.15) | (-0.03 - 0.09) | (-0.02 - 0.12) |
| Alcohol intake: - Several times per week (Reference category: Daily) | 0.03 | 0.03 | -0.20+ | 0.03 | 0.03 | 0.03 | 0.02 | 0.01 |
|  | (-0.04 - 0.09) | (-0.04 - 0.10) | (-0.42 - 0.01) | (-0.04 - 0.09) | (-0.04 - 0.10) | (-0.04 - 0.09) | (-0.04 - 0.08) | (-0.05 - 0.07) |
| - Once a week | 0.06+ | 0.07+ | -0.10 | 0.06+ | 0.06+ | 0.06+ | 0.04 | 0.05 |
|  | (-0.01 - 0.13) | (-0.00 - 0.15) | (-0.33 - 0.13) | (-0.01 - 0.13) | (-0.01 - 0.14) | (-0.01 - 0.13) | (-0.02 - 0.10) | (-0.02 - 0.12) |
| - 1-3 times a month | 0.05 | 0.05 | -0.00 | 0.05 | 0.06 | 0.05 | 0.04 | 0.05 |
|  | (-0.02 - 0.13) | (-0.03 - 0.13) | (-0.25 - 0.25) | (-0.02 - 0.13) | (-0.02 - 0.13) | (-0.02 - 0.13) | (-0.02 - 0.11) | (-0.03 - 0.12) |
| - Less often | 0.08* | 0.07+ | -0.06 | 0.08* | 0.08* | 0.08* | 0.06* | 0.07* |
|  | (0.01 - 0.15) | (-0.00 - 0.15) | (-0.28 - 0.15) | (0.01 - 0.15) | (0.01 - 0.15) | (0.01 - 0.15) | (0.00 - 0.12) | (0.00 - 0.14) |
| - Never | 0.06 | 0.07 | -0.16 | 0.06 | 0.06 | 0.06 | 0.04 | 0.05 |
|  | (-0.03 - 0.15) | (-0.03 - 0.17) | (-0.39 - 0.08) | (-0.03 - 0.15) | (-0.03 - 0.15) | (-0.03 - 0.15) | (-0.03 - 0.12) | (-0.03 - 0.13) |
| Number of chronic conditions | 0.05*** | 0.04*** | 0.04** | 0.05*** | 0.05*** | 0.05*** | 0.02*** | 0.03*** |
|  | (0.04 - 0.06) | (0.03 - 0.06) | (0.01 - 0.07) | (0.04 - 0.06) | (0.04 - 0.06) | (0.04 - 0.06) | (0.01 - 0.03) | (0.02 - 0.04) |
| Physical functioning | -0.00* | -0.00** | 0.00 | -0.00* | -0.00* | -0.00* | -0.00 | 0.00 |
|  | (-0.00 - -0.00) | (-0.00 - -0.00) | (-0.00 - 0.00) | (-0.00 - -0.00) | (-0.00 - -0.00) | (-0.00 - -0.00) | (-0.00 - 0.00) | (-0.00 - 0.00) |
| Probable depression: Yes (Reference category: No) | 0.34*** | 0.36*** | 0.45*** | 0.34*** | 0.34*** | 0.34*** | 0.14** | 0.25*** |
|  | (0.25 - 0.44) | (0.25 - 0.48) | (0.22 - 0.68) | (0.25 - 0.44) | (0.25 - 0.44) | (0.25 - 0.44) | (0.05 - 0.23) | (0.15 - 0.34) |
| Self-esteem |  |  |  |  |  |  | -0.63*** |  |
|  |  |  |  |  |  |  | (-0.92 - -0.34) |  |
| Self-efficacy |  |  |  |  |  |  |  | -0.32* |
|  |  |  |  |  |  |  |  | (-0.61 - -0.04) |
| Individuals | 3,059 | 2,544 | 406 | 3,059 | 3,059 | 3,059 | 3,059 | 3,058 |
| R² | 0.11 | 0.10 | 0.13 | 0.11 | 0.11 | 0.11 | 0.33 | 0.23 |

Supplementary Table 2. Professional prestige and social isolation. Results of linear regressions (wave 8) (all covariates are displayed; interaction terms are also shown)

|  |  |  |  |  |  |  |  |  |
| --- | --- | --- | --- | --- | --- | --- | --- | --- |
|  | Social isolation | Social isolation | Social isolation | Social isolation | Social isolation | Social isolation | Social isolation | Social isolation |
|  |  |  |  |  |  |  |  |  |
|  |  |  |  |  |  |  |  |  |
| Professional prestige for respondents | -0.03** |  |  | -0.04** | -0.02 | -0.11 | -0.07 | 0.04 |
|  | (-0.06 - -0.01) |  |  | (-0.07 - -0.01) | (-0.16 - 0.12) | (-0.29 - 0.08) | (-0.24 - 0.11) | (-0.12 - 0.20) |
|  |  |  |  |  |  |  |  |  |
| Professional prestige for partners |  | -0.02 |  |  |  |  |  |  |
|  |  | (-0.04 - 0.01) |  |  |  |  |  |  |
| Professional prestige for last spouses (when respondents are currently without a partner) |  |  | -0.03 |  |  |  |  |  |
|  |  |  | (-0.09 - 0.04) |  |  |  |  |  |
|  |  |  |  |  |  |  |  |  |
| Interaction terms: |  |  |  |  |  |  |  |  |
|  |  |  |  |  |  |  |  |  |
| Women x Professional prestige for respondents |  |  |  | 0.02 |  |  |  |  |
|  |  |  |  | (-0.03 - 0.06) |  |  |  |  |
| Age x Professional prestige for respondents |  |  |  |  | 0.00 |  |  |  |
|  |  |  |  |  | (-0.00 - 0.00) |  |  |  |
| Medium education x Professional prestige for respondents |  |  |  |  |  | 0.06 |  |  |
|  |  |  |  |  |  | (-0.13 - 0.24) |  |  |
| High education x Professional prestige for respondents |  |  |  |  |  | 0.09 |  |  |
|  |  |  |  |  |  | (-0.10 - 0.27) |  |  |
| Self-esteem x Professional prestige for respondents |  |  |  |  |  |  | 0.01 |  |
|  |  |  |  |  |  |  | (-0.04 - 0.06) |  |
| Self-efficacy x Professional prestige for respondents |  |  |  |  |  |  |  | -0.02 |
|  |  |  |  |  |  |  |  | (-0.07 - 0.03) |
| Covariates |  |  |  |  |  |  |  |  |
|  |  |  |  |  |  |  |  |  |
| Age | -0.00 | -0.00 | -0.00 | -0.00 | -0.00 | -0.00 | -0.00 | -0.00* |
|  | (-0.01 - 0.00) | (-0.00 - 0.00) | (-0.01 - 0.01) | (-0.01 - 0.00) | (-0.01 - 0.01) | (-0.01 - 0.00) | (-0.00 - 0.00) | (-0.01 - -0.00) |
| Sex: Women (Reference category: Men) | -0.03 | -0.02 | -0.05 | -0.12 | -0.03 | -0.03 | 0.02 | -0.03 |
|  | (-0.07 - 0.02) | (-0.06 - 0.03) | (-0.18 - 0.09) | (-0.39 - 0.15) | (-0.07 - 0.01) | (-0.07 - 0.01) | (-0.01 - 0.06) | (-0.07 - 0.01) |
| Marital status: - Married, living separated from spouse (Reference category: Married, living together with spouse) | 0.12+ | 0.19+ |  | 0.12+ | 0.12+ | 0.12+ | -0.03 | 0.07 |
|  | (-0.02 - 0.26) | (-0.01 - 0.40) |  | (-0.02 - 0.26) | (-0.02 - 0.26) | (-0.02 - 0.26) | (-0.15 - 0.10) | (-0.04 - 0.19) |
| - Divorced | 0.05 | -0.01 | -0.02 | 0.05 | 0.05 | 0.05 | 0.05+ | 0.06+ |
|  | (-0.02 - 0.11) | (-0.10 - 0.09) | (-0.25 - 0.20) | (-0.02 - 0.11) | (-0.02 - 0.11) | (-0.02 - 0.11) | (-0.00 - 0.11) | (-0.00 - 0.12) |
| - Widowed | 0.01 | -0.05 | -0.04 | 0.01 | 0.01 | 0.01 | 0.01 | 0.03 |
|  | (-0.06 - 0.07) | (-0.16 - 0.05) | (-0.26 - 0.18) | (-0.06 - 0.07) | (-0.06 - 0.07) | (-0.06 - 0.07) | (-0.05 - 0.06) | (-0.03 - 0.09) |
| - Single | 0.12** | 0.12* | -0.16 | 0.12** | 0.12** | 0.12** | 0.03 | 0.07+ |
|  | (0.04 - 0.20) | (0.01 - 0.22) | (-0.56 - 0.23) | (0.04 - 0.20) | (0.04 - 0.20) | (0.04 - 0.20) | (-0.04 - 0.09) | (-0.00 - 0.14) |
| Employment status: - Retired (Reference category: Employed) | 0.02 | -0.00 | -0.02 | 0.02 | 0.02 | 0.02 | 0.01 | 0.02 |
|  | (-0.04 - 0.09) | (-0.07 - 0.06) | (-0.23 - 0.19) | (-0.04 - 0.09) | (-0.04 - 0.09) | (-0.04 - 0.09) | (-0.04 - 0.06) | (-0.04 - 0.08) |
| - Other: not employed | 0.07 | 0.02 | 0.06 | 0.07 | 0.07 | 0.07 | 0.04 | 0.08 |
|  | (-0.03 - 0.18) | (-0.08 - 0.13) | (-0.25 - 0.37) | (-0.03 - 0.18) | (-0.03 - 0.18) | (-0.03 - 0.18) | (-0.05 - 0.13) | (-0.02 - 0.18) |
| Education: - Medium education (Reference category: Low education) | 0.01 | -0.08 | 0.25 | 0.00 | 0.01 | -0.28 | 0.08 | 0.03 |
|  | (-0.13 - 0.14) | (-0.23 - 0.08) | (-0.08 - 0.59) | (-0.13 - 0.14) | (-0.13 - 0.14) | (-1.27 - 0.72) | (-0.04 - 0.19) | (-0.09 - 0.15) |
| - High education | -0.03 | -0.14+ | 0.20 | -0.03 | -0.03 | -0.50 | 0.08 | 0.00 |
|  | (-0.16 - 0.11) | (-0.29 - 0.02) | (-0.15 - 0.54) | (-0.17 - 0.10) | (-0.16 - 0.11) | (-1.49 - 0.49) | (-0.03 - 0.20) | (-0.12 - 0.13) |
| Frequency of sports activities: - Several times per week (Reference category: Daily) | 0.01 | 0.00 | 0.04 | 0.01 | 0.01 | 0.01 | 0.01 | -0.00 |
|  | (-0.05 - 0.08) | (-0.07 - 0.08) | (-0.13 - 0.22) | (-0.06 - 0.08) | (-0.05 - 0.08) | (-0.06 - 0.08) | (-0.04 - 0.06) | (-0.06 - 0.06) |
| - Once a week | 0.05 | 0.00 | 0.09 | 0.05 | 0.05 | 0.05 | 0.01 | 0.03 |
|  | (-0.02 - 0.12) | (-0.08 - 0.08) | (-0.11 - 0.29) | (-0.02 - 0.12) | (-0.02 - 0.12) | (-0.02 - 0.12) | (-0.05 - 0.07) | (-0.04 - 0.09) |
| - 1-3 times a month | 0.01 | -0.07 | 0.19 | 0.01 | 0.01 | 0.01 | -0.00 | 0.00 |
|  | (-0.09 - 0.10) | (-0.16 - 0.03) | (-0.11 - 0.48) | (-0.09 - 0.10) | (-0.09 - 0.10) | (-0.09 - 0.10) | (-0.08 - 0.07) | (-0.09 - 0.09) |
| - Less often | 0.03 | -0.02 | 0.23 | 0.03 | 0.03 | 0.03 | 0.00 | 0.02 |
|  | (-0.06 - 0.11) | (-0.12 - 0.07) | (-0.06 - 0.51) | (-0.06 - 0.11) | (-0.06 - 0.11) | (-0.06 - 0.11) | (-0.07 - 0.07) | (-0.05 - 0.10) |
| - Never | 0.07+ | 0.04 | 0.15 | 0.07+ | 0.07+ | 0.07+ | 0.02 | 0.04 |
|  | (-0.01 - 0.14) | (-0.05 - 0.12) | (-0.04 - 0.34) | (-0.01 - 0.14) | (-0.01 - 0.14) | (-0.01 - 0.14) | (-0.04 - 0.09) | (-0.03 - 0.11) |
| Alcohol intake: - Several times per week (Reference category: Daily) | 0.03 | 0.04 | -0.33* | 0.03 | 0.03 | 0.03 | 0.03 | 0.01 |
|  | (-0.04 - 0.10) | (-0.04 - 0.11) | (-0.59 - -0.08) | (-0.04 - 0.11) | (-0.04 - 0.10) | (-0.04 - 0.10) | (-0.03 - 0.08) | (-0.05 - 0.08) |
| - Once a week | 0.05 | 0.05 | -0.08 | 0.05 | 0.05 | 0.05 | 0.03 | 0.04 |
|  | (-0.02 - 0.13) | (-0.02 - 0.13) | (-0.36 - 0.19) | (-0.02 - 0.13) | (-0.02 - 0.13) | (-0.02 - 0.13) | (-0.04 - 0.09) | (-0.03 - 0.11) |
| - 1-3 times a month | 0.08+ | 0.05 | -0.10 | 0.08+ | 0.08+ | 0.08+ | 0.07* | 0.07+ |
|  | (-0.00 - 0.16) | (-0.04 - 0.13) | (-0.37 - 0.17) | (-0.00 - 0.16) | (-0.00 - 0.16) | (-0.00 - 0.16) | (0.00 - 0.13) | (-0.01 - 0.14) |
| - Less often | 0.10* | 0.09* | -0.15 | 0.10* | 0.10* | 0.10* | 0.08* | 0.09* |
|  | (0.02 - 0.18) | (0.01 - 0.17) | (-0.41 - 0.10) | (0.02 - 0.18) | (0.02 - 0.18) | (0.02 - 0.18) | (0.02 - 0.15) | (0.02 - 0.16) |
| - Never | 0.09+ | 0.09+ | -0.15 | 0.09+ | 0.09+ | 0.09+ | 0.07+ | 0.08+ |
|  | (-0.01 - 0.18) | (-0.01 - 0.20) | (-0.44 - 0.14) | (-0.01 - 0.18) | (-0.01 - 0.18) | (-0.01 - 0.18) | (-0.01 - 0.15) | (-0.01 - 0.16) |
| Number of chronic conditions | 0.06*** | 0.05*** | 0.05* | 0.06*** | 0.06*** | 0.06*** | 0.02*** | 0.04*** |
|  | (0.04 - 0.07) | (0.04 - 0.06) | (0.01 - 0.08) | (0.04 - 0.07) | (0.04 - 0.07) | (0.04 - 0.07) | (0.01 - 0.03) | (0.03 - 0.05) |
| Physical functioning | -0.00*** | -0.00*** | -0.00 | -0.00*** | -0.00*** | -0.00*** | -0.00* | -0.00 |
|  | (-0.00 - -0.00) | (-0.00 - -0.00) | (-0.00 - 0.00) | (-0.00 - -0.00) | (-0.00 - -0.00) | (-0.00 - -0.00) | (-0.00 - -0.00) | (-0.00 - 0.00) |
| Probable depression: Yes (Reference category: No) | 0.46*** | 0.47*** | 0.44** | 0.46*** | 0.46*** | 0.46*** | 0.22*** | 0.35*** |
|  | (0.35 - 0.57) | (0.34 - 0.60) | (0.18 - 0.71) | (0.35 - 0.57) | (0.35 - 0.57) | (0.35 - 0.57) | (0.13 - 0.31) | (0.25 - 0.45) |
| Self-esteem |  |  |  |  |  |  | -0.86*** |  |
|  |  |  |  |  |  |  | (-1.16 - -0.57) |  |
| Self-efficacy |  |  |  |  |  |  |  | -0.38* |
|  |  |  |  |  |  |  |  | (-0.68 - -0.08) |
| Individuals | 3,060 | 2,536 | 409 | 3,060 | 3,060 | 3,060 | 3,060 | 3,059 |
| R² | 0.14 | 0.14 | 0.13 | 0.14 | 0.14 | 0.14 | 0.41 | 0.27 |
